# Connectivity in grey reef sharks (*Carcharhinus amblyrhynchos*) determined using empirical and simulated genetic data

**DOI:** 10.1038/srep13229

**Published:** 2015-08-28

**Authors:** Paolo Momigliano, Robert Harcourt, William D. Robbins, Adam Stow

**Affiliations:** 1 Department of Biological Sciences, Macquarie University, Sydney, 2109 New South Wales, Australia; 2 Sydney Institute of Marine Science, 19 Chowder Bay Road, Mosman, 2088 New South Wales, Australia; 3 College of Marine and Environmental Science, James Cook University, Townsville, 4810 Queensland, Australia; 4 Wildlife Marine, Perth, 6020 Western Australia, Australia

## Abstract

Grey reef sharks (*Carcharhinus amblyrhynchos*) can be one of the numerically dominant high order predators on pristine coral reefs, yet their numbers have declined even in the highly regulated Australian Great Barrier Reef (GBR) Marine Park. Knowledge of both large scale and fine scale genetic connectivity of grey reef sharks is essential for their effective management, but no genetic data are yet available. We investigated grey reef shark genetic structure in the GBR across a 1200 km latitudinal gradient, comparing empirical data with models simulating different levels of migration. The empirical data did not reveal any genetic structuring along the entire latitudinal gradient sampled, suggesting regular widespread dispersal and gene flow of the species throughout most of the GBR. Our simulated datasets indicate that even with substantial migrations (up to 25% of individuals migrating between neighboring reefs) both large scale genetic structure and genotypic spatial autocorrelation at the reef scale were maintained. We suggest that present migration rates therefore exceed this level. These findings have important implications regarding the effectiveness of networks of spatially discontinuous Marine Protected Areas to protect reef sharks.

The conservation of higher order predators is important for the maintenance of marine biodiversity, due to their potentially pivotal role in shaping communities^1^. Predator removal may have far reaching consequences on lower trophic levels^2^, including cascading effects on both abundance^2^,^3^ and behavior^4^. This can reduce ecosystem resilience to natural and anthropogenic disturbances^5^ and in extreme cases lead to ecosystem phase-shifts^6^. The grey reef shark, *Carcharhinus amblyrhynchos*, the whitetip reef shark, *Triaenodon obesus* and the blacktip reef shark, *C. melanopterus* are abundant mesopredators that occupy the highest trophic level of permanent food webs in coral reefs^7^,^8^. Recently it has been shown that pristine coral reefs have high biomasses of higher order predators, and reef sharks can make up the majority of that biomass^9^,^10^,^11^. Their potential loss is therefore a major threat to the long term resilience of coral reef ecosystems.

Reef sharks exhibit a combination of life history strategies and habitat specialization that make them particularly susceptible to anthropogenic pressure. They are slow growing, have delayed sexual maturity, and low reproductive output^12^,^13^ and are therefore susceptible to even modest levels of fishing pressure^14^. As most species are strictly associated with coral reefs^15^,^16^, they are further threatened by the ongoing loss of coral reef habitat^17^ due to the synergistic effects of overfishing^18^, eutrophication^19^, ocean acidification^20^ and increased sea surface temperatures^21^. Not surprisingly, their numbers have been shrinking over recent decades^14^,^22^,^23^,^24^, particularly in areas open to fisheries, raising concerns about the long-term viability of reef shark populations, even in highly regulated marine parks such as the Great Barrier Reef Marine Park (GBR), Australia^14^,^24^.

The grey reef shark is the most abundant reef shark in the Indo-Pacific, accounting for up to 50% of higher order predator biomass on healthy reefs^9^. Within the GBR the grey reef shark is the most threatened by fishing pressure, having the fastest rate of decline of all reef shark species^14^,^24^. It has been suggested that grey reef sharks are protected when they inhabit very small but strictly enforced MPAs, where their density can be an order of magnitude higher than in neighboring areas open to fisheries^23^,^24^,^25^. However, acoustic telemetry studies show that grey reef sharks may exhibit limited site fidelity, with some individuals detected transiting across multiple reefs, repeatedly crossing the boundaries of different management zones^26^,^27^.

To be effective in increasing local biomass of target species, MPAs must meet two requirements: 1) they need to protect the target species by encompassing a large proportion of its home range; and 2) they need to be at least partially self-seeding (animals within an MPA need to contribute to the next generation)^28^,^29^. In this study we use empirical and simulated genetic data to investigate gene-flow for the grey reef shark within the GBR. We hypothesize that if grey reef shark reef fidelity is high, patterns of genetic structure will arise over large distances. Similarly, if self-recruitment is high, patterns of genotypic spatial autocorrelation should be detected i.e. genetic distance between individuals should increase with geographic distance.

## Results

### Diversity indices

**Figure 1:**
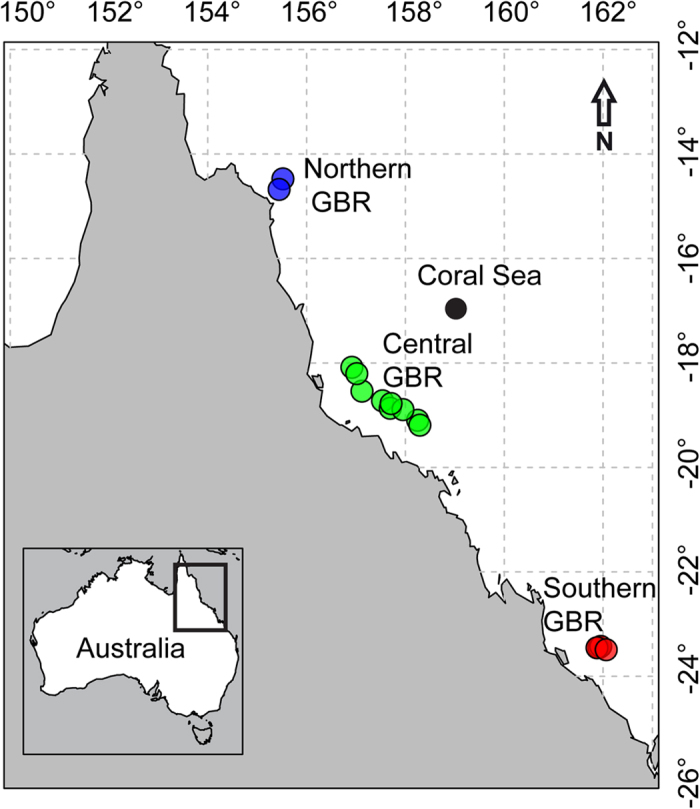
Sampling design. Genetic samples were collected from 121 grey reef sharks in three distinct regions of the GBR and the Coral Sea (Herald Cays). In the North GBR, 32 individuals were sampled from 2 reefs; in the Central GBR, 54 individuals from 9 reefs and in the southern GBR we sampled 27 individuals from 3 reefs. Furthermore, 8 individuals were sampled from the Herald Cays in the Coral Sea. The map was produced using the package “maps” in the R statistical environment^75^.

Genetic data were obtained for a total of 121 grey reef sharks sampled along a latitudinal gradient spanning more than 1200 km and 9° of latitude (23° to 14° S, Fig. 1, Supplementary Table S1) within the GBR. We genotyped all individuals at 16 microsatellite loci. Overall diversity indices for the microsatellite dataset are given in the supplementary materials (Supplementary Table S2), and additional diversity indices for each region are given in Table 1. The number of alleles at each locus ranged from 3 to 34, observed heterozygosity for each locus ranged from 0.356 to 0.958 (Supplementary Table S2). None of the loci significantly deviated from Hardy-Weinberg Equilibrium (HWE), and there was no evidence of Linkage Disequilibrium (LD). Within each region, allelic richness (A_r_) ranged from 5.6 to 6.93, and observed heterozygosity over all loci ranged from 0.79 to 0.8 (Table 1). Fixation indices (*F*_is_) were small or negative (Table 1) and non-significant (95% confidence intervals overlapped with 0). We obtained sequence data for 813 bp of the ND4 gene from 109 individuals (Table 1, GenBank accession numbers KT326195-KT326303). A total of 10 polymorphic sites were present, of which 7 were parsimony informative (3 singletons). Ten distinct haplotypes were identified, and haplotype diversity (*h*) ranged from 0.61 to 0.8 (Table 1).

### Spatial structure analyses

**Figure 2:**
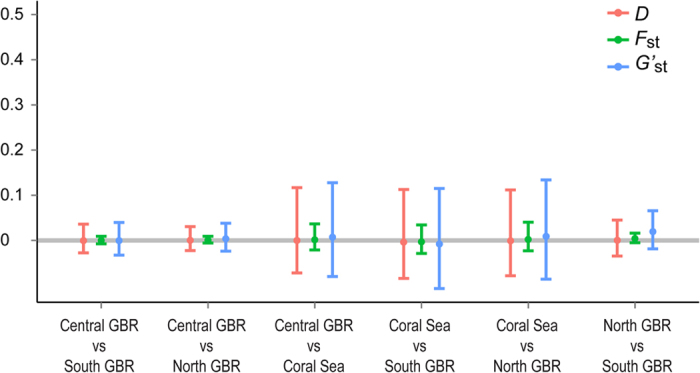
Between-region estimates of genetic differentiation with 95% confidence intervals: Weir and Cockerham *F*_st_ (*F*_st_), Hedrick’s *G’*_st_ (*G’*_st_) and Jost’s *D* (*D*). All estimates were very close to 0 and non-significant. Confidence intervals were estimated using 1000 bootstrap pseudo-replicates.

**Figure 3:**
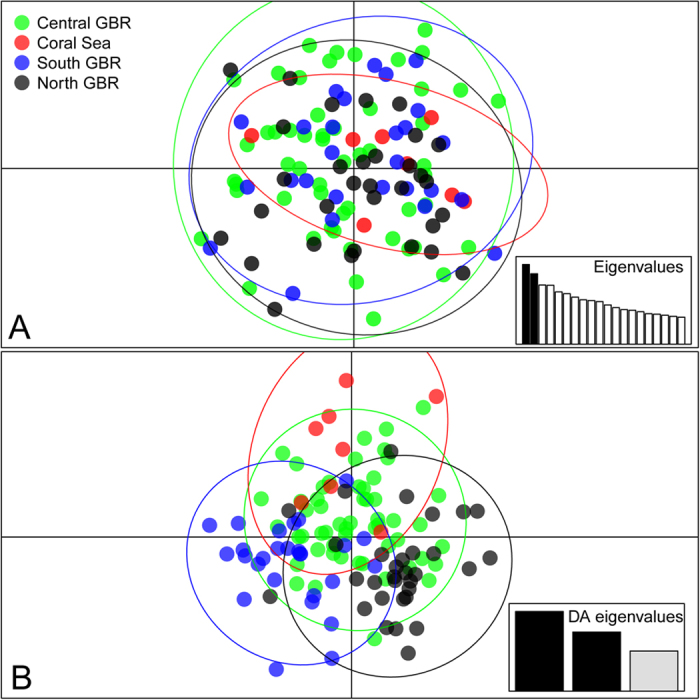
Results from PCA (A) and DAPC (B) analyses. Groups were defined as geographic regions within the sampling area (North GBR, Central GBR, South GBR and Coral Sea). Eigenvalues representing the variance explained by principal components (**A**) and discriminant factors (**B**) are shown in the scree plots. The x and y axes represent, respectively, the first and second principal components (**A**) and the first and second discriminant factors (**B**). Individuals are represented by dots, groups are color-coded and depicted by 95% inertia ellipses.

Pairwise *F*_st_ estimates between regions for the mtDNA dataset were small (<0.015) and did not significantly differ from 0 (p > 0.27). Similarly, all measures of genetic differentiation obtained from the microsatellite dataset were not significantly different from 0 (Fig. 2). For the whole dataset Meirman’s *F’*_*s*t_ was very small (0.003) and non-significant (p > 0.3). The results from the PCA analysis (Fig. 3A) revealed no difference in overall genetic variance among regions; the 95% confidence ellipses of each region were clearly overlapping, and densities along the first two principal components were very similar (data not shown). Even the Discriminant Analysis of Principal Components (DAPC), which is widely used to resolve weak genetic structure^30^,^31^, failed to reveal any clear partitioning between regions (Fig. 3B).Together these results are strong evidence of extensive gene flow over the entire sampling area.

Spatial autocorrelation analyses did not reveal any spatial structure regardless of whether the dataset was analysed as a whole, or split according to sex (Supplementary Fig. S1). Autocorrelation coefficients (*r*) were very close to 0 and their 95% confidence interval always overlapped with 0 and fell well within the expectations of the null model. None of the correlograms were statistically significant according to the non-parametric heterogeneity test developed by Smouse, *et al.*^32^ (p > 0.1). This complete lack of spatial autocorrelation suggests that reef fidelity persisting for generations is unlikely and is consistent with the results from between region comparisons for both the mtDNA and microsatellite datasets.

### Simulations

**Figure 4:**
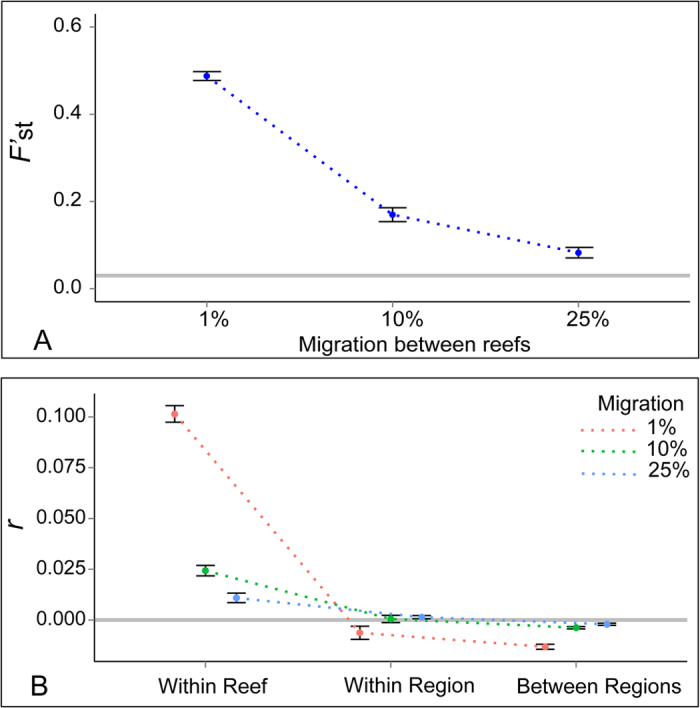
Results from the simulated datasets under different migration scenarios. Top graph (**A**) shows estimated global Meirman’ s *F’*_st_ estimates between regions. Bottom graph (**B**) shows spatial autocorrelation estimates (*r* across different spatial scales: within reef, within region and across the entire simulated seascape). All scenarios resulted in positive and significant patterns of spatial autocorrelation. Error bars represent 95% confidence intervals obtained from 10 independent simulations. Grey lines in both (**A,B**) represent the estimates obtained from the empirical dataset.

In contrast with the results from the empirical microsatellite and mitochondrial datasets, genetic differentiation between regions was clearly evident in the simulations, even when migration between neighbouring reefs was set at 25% (Fig. 4A). Estimates of overall Meirman’s *F’*_st_ range from 0.49 (1% migration) to 0.08 (25% migration), were all significant (p < 0.05) and their confidence interval did not overlap with the *F’*_st_ value for the observed data. Positive spatial autocorrelation was detected in all simulations, and the extent of spatial autocorrelation was largely dependent on migration rates (Fig. 4B). Positive spatial-autocorrelation was detected in our simulations despite the fact that simulation parameters were overly conservative, and that we used smaller sample sizes than in our empirical dataset (see Supplementary Materials). These results suggest that the lack of spatial structure observed in the empirical dataset is not the result of limited analytical power, and likely reflects very high levels of connectivity.

## Discussion

This first assessment of genetic connectivity in grey reef sharks was carried out across a latitudinal gradient spanning more than 1200 km and 9° of latitude i.e. 2/3 of the entire length of the Great Barrier Reef. Despite this spatial scale, we found no evidence of genetic differentiation across the study area and no evidence of fine-scale spatial structure. Our simulated data shows that we had the statistical power to detect structure at the regional geographical scale as well as fine scale spatial autocorrelation, even assuming high migration rates (~25% of individuals) between neighbouring reefs. With respect to temporal scales relevant to ecological processes, we found no genotypic spatial autocorrelation within reefs. Structuring of allele frequencies via genetic drift may be effectively prevented by a small number of migrants per generation in large populations, and therefore may only become evident across large spatial and temporal scales^33^. It is possible therefore that large scale dispersal by a portion of the migrants resulted in an overestimate of genetic drift in our simulations. Genotypic spatial autocorrelation on the other hand reflects individual dispersal across a few generations^34^, is most pronounced at small spatial scales, measurable within less than 10 generations and reaches quasi-stationarity within 50–100 generations^35^. We can therefore conclude that the lack of spatial autocorrelation reflects sufficient dispersal over a few generations to prevent genotypic structure from accumulating within the GBR for grey reef sharks.

Our results have important implications for the management of this species, and are in sharp contrast with the findings of several previous studies on other reef associated sharks^36^,^37^,^38^,^39^. In the closely related *Carcharhinus melanopterus* there are clear patterns of genetic structure between reefs separated by as little as 50 km within French Polynesia^36^,^37^. Similarly, a recent study on gene flow of the other principal reef associated carcharhinid *(Triaenodon obesus*), also revealed very strong genetic structure (Φ_st _= 0.25) between North and Central GBR^38^. Perhaps more surprising than the lack of spatial structure within the GBR is an absence of genetic partitioning between the GBR and the Coral Sea (Herald Cays). The Herald Cays are isolated coral reefs located approximately 200 km east of the edge of the continental shelf where the outer reefs of the GBR lie. They are separated from the GBR by waters deeper than 1000 m. Therefore, it is evident that grey reef sharks not only disperse between semi isolated reefs within the GBR, but have the capacity for long distance dispersal across oceanic waters. Heupel, *et al.*^26^ reported that in June 2008 an individual grey reef shark undertook a large scale movement (124 km) from Osprey Reef in the Coral Sea to the Ribbon Reefs in the North GRB. This same individual returned to Osprey reef in October of that year^40^. Our results concord with these observations and suggest that movements on the scale of 100 s of km across oceanic waters occur at a rate sufficient to prevent detectable genetic drift between isolated reefs in the Coral Sea and the GBR.

Grey reef sharks are often described as strict habitat specialists, associated exclusively with coral reef habitats^15^,^17^,^25^. Previous telemetry studies suggested that grey reef sharks exhibit a high degree of site fidelity in isolated seamounts, as most animals show year-round residency within the study area^40^,^41^. This has led to the presumption that even within the GBR grey reef sharks will exhibit a high degree of reef fidelity, and therefore will be well protected by strictly enforced but small marine protected areas^23^,^24^. This interpretation has been supported by robust estimates of grey reef shark abundances across different management zones, showing that grey reef shark densities are up to an order of magnitude higher in strictly enforced no access areas compared to areas open to fisheries^23^,^24^. Robbins, *et al.*^24^ and Rizzari, *et al.*^23^ concluded that differences in fishing mortality are the main cause of the observed differences, and that therefore even very small MPAs can be very effective in increasing grey reef shark biomass if they are well complied with. In support, Hisano, *et al.*^14^ argued that quantitative projections of abundances in fished versus unfished reefs calculated from differences in fishing mortality are also compatible with the observed differences.

This interpretation, however, relies on the assumption that grey reef sharks within each reef represent a closed population^14^. That is, it assumes: 1) that grey reef sharks are protected from fishing throughout most of their home range, and 2) that reef sharks within each MPA contribute to the biomass of future generations within the same MPA. Telemetry studies carried out in the same regions do not support the first assumption. Heupel, *et al.*^26^ found that in the North GBR, grey reef sharks show little fidelity to the reef where they were initially tagged, consistently moving across different management zones. Even in the Central GBR, where reefs are isolated by much larger distances, 40% of the grey reef sharks (90% of adult males) were recorded moving between reefs, often crossing different management zones in a period of less than 2 years^27^. Furthermore we found no evidence of genetic spatial autocorrelation, suggesting that that migration rates between neighbouring reefs likely exceed 25%.

Heupel, *et al.*^26^ suggested that differences in fishing mortality may not be the only driver of the differences in abundances observed by Robbins, *et al.*^24^, and that other factors, such as differences in carrying capacity between areas open to fisheries and protected areas, might also be at play. It is possible that additional factors may affect grey reef shark abundances in strictly enforced protected areas. For example, strictly enforced MPAs have much larger fish biomasses^42^ compared to areas open to fisheries, and therefore could potentially sustain higher predator abundances. Espinoza, *et al.*^25^ also found a positive effect of hard coral cover on grey reef shark abundance. Reefs with high hard coral cover provide higher structural complexity, and this in turn may sustain a higher biomass^43^ of potential prey. It is well established that MPAs that have been enforced for extended periods of times are more effective at preventing losses in hard coral cover^44^. In the GBR, no-take areas have on average higher coral cover than areas open to fisheries^45^, and experience much lower frequencies of crown-of-thorns starfish (*Acanthaster planci*) outbreaks^46^, a leading cause of decline in coral cover in the region^47^.

The lack of genetic structure in this study may be the result of either high juvenile or adult dispersal, or both, albeit available movement data point to adults representing the dispersal stage. In two acoustic tagging studies conducted on the GBR, adult grey reef sharks were found to exhibit limited reef fidelity and move repeatedly between multiple reefs. Espinoza, *et al.*^27^ reported that approximately 90% of adult males were detected on multiple (up to five), semi-isolated reefs in the Central GBR, while only one out of 12 tracked juveniles was detected at more than one reef. Heupel, *et al.*^26^ also found that small juveniles exhibited high reef fidelity, while adults move continuously between reefs. Espinoza, *et al.*^48^ found that larger grey reef sharks and silvertip sharks (*Carcharhinus albimarginatus*), and especially large males, have wider home ranges that encompass multiple reefs. Given that larger sharks can traverse greater distances for comparable energetic expenditure^49^, and are less vulnerable to predation, increased dispersal by adults is not surprising. However, a closely related species (*C. melanopterus*) has been shown to exhibit ontogenetic dispersal between inshore nursery and offshore reefs^50^. While there is no empirical evidence of the existence of nursery areas for grey reef sharks (juveniles co-occur with adults on coral reefs), the hypothesis that ontogenetic dispersal occurs cannot be discarded. Future genetic studies could compare genetic structure in different life-stages (juveniles, sub adults and adults), to test ontogenetic dispersal differences.

Espinoza, *et al.*^27^ suggested that in grey reef sharks high adult male dispersal may provide benefits in terms of genetic and demographic connectivity and/or provide foraging opportunities while reducing competition with large resident females. Our data do not show any evidence of sex-bias in dispersal, however these findings do not necessarily refute the hypothesis of sex-bias dispersal if we assume female migration was sufficient to prevent any detectable effect of genetic drift. Espinoza, *et al.*^27^ found that >25% of mature females were detected on two distinct reefs within a period of 2 years. Since they investigated movement patterns between semi-isolated reefs (10 s of km apart) within the Central GBR, it is logical to assume that in other regions of the GBR where reefs are less isolated, movements between reefs would occur at higher frequencies, given that reefs that are separated by small distances may be perceived as a continuous habitat^26^,^29^. It therefore seems feasible, particularly given the results of our simulations, that female dispersal is large enough to mask any effect of sex bias dispersal at the spatial scale of this study.

The widespread gene flow documented in this study, along with the findings of recent acoustic telemetry studies on grey reef shark movement suggest that while MPAs play an important role in protecting grey reef sharks, they are no panacea for reef shark conservation. Complementary management strategies appear necessary. The shortcomings of the current model of discontinuous networks of MPAs for the protection of coral reef organisms with adult dispersal have recently been highlighted by Momigliano, *et al.*^29^ and Espinoza, *et al.*^48^. Potential solutions that have been proposed include the design of MPAs which include closely spaced (<20 km apart) reefs^48^ and the establishment of protected (i.e. no fishing) connectivity corridors that provide for movements between MPAs, thereby extending protection for animals with larger home ranges^29^. Furthermore, complementary management strategies based on fishery restrictions, such the ones implemented in eastern Australia in 2009, appear necessary.

## Material and Methods

### Sample collection

Fin clips from 121 grey reef sharks were obtained along a latitudinal gradient spanning more than 1200 km and 9° of latitude (23° to 14° S, Fig. 1) within the GBR. Specimens from the North GBR (2 reefs, N = 32), the Central GBR (9 reefs, N = 54), the Coral Sea (1 reef, N = 8) and the South GBR (1 reef, N=2) were collected between February 2001 and April 2005 and represent a subsample from Robbins^12^. The remaining individuals from the South GBR (2 reefs, N = 25) were collected in April 2013 by hook and line. Sharks were captured in shallow waters (<30 m) on the reef slopes of Wistari Reef and Heron Island reef using heavy handlines and 16/0 tuna circle hooks to reduce the risk of deep hooking. A fin clip of approximately 15 mm^2^ was taken before the animals were released. Sampling was conducted in accordance with Macquarie University’s approved guidelines and the sampling protocol was approved by Macquarie University’s Animal Ethics Committee (AEC reference No.: 2012/044). Samples were collected under permit G13/35796.1 issued by the Great Barrier Reef Marine Park Authority (GBRMPA). Samples were stored in 95% ethanol at −20 °C. Details of sampling, including geographic coordinates of all sampling locations, are given in the supplementary materials (Supplementary Table S1).

### Laboratory procedures

DNA was extracted from fin clips of 121 grey reef sharks using a modified Chelex protocol^51^. A fragment of the NADH deydrogenase subunit 4 (ND4) gene approximately 850 bp long was amplified by Polymerase Chain Reaction (PCR) using the primers ND4^52^ and H12293-Leu^53^. We amplified a suite of 15 microsatellite loci recently isolated for the grey reef sharks by Momigliano, *et al.*^54^ and an additional microsatellite locus (Cpl169) which was originally developed for *C. plumbeus* by Portnoy, *et al.*^55^. The 16 microsatellite loci used are listed in the supplementary materials (Supplementary Table S2).

### Empirical Data Analysis

Mitochondrial DNA sequences were aligned using Bioedit^56^. Molecular indices, including haplotype diversity (*h*) and nucleotide diversity (π) were estimated in DNAsp^57^. Pairwise *F*_st_ between regions based on ND4 gene sequences were calculated in Arlequin 3.5^58^.

Microsatellites were scored using GeneMapper (Version 4), and alleles were binned using the software TANDEM following the method outlined by Matschiner and Salzburger^59^. The dataset was tested for significant departures from HWE in Genepop 4.2^60^ and for the presence of null alleles using the software MICROCHECKER^61^. Tests for LD were carried out using the software FSTAT version 2.9.3.2^62^, using a sequential Holm-Bonferroni correction. Because there was no statistically significant departure from HWE and no evidence of linkage disequilibrium, all loci were used in the analyses. The microsatellite dataset is publicly accessible through the Dryad repository (doi:10.5061/dryad.362s5).

Allele frequencies and measures of genetic differentiation were estimated in the R statistical environment using the package diveRsity^63^. Different measures of genetic differentiation (Weir and Cockerham *F*_st_^64^, Hedrick’s *G’*_*st*_^65^, and Jost’s *D*^66^), and their 95% confidence intervals were estimated using 1000 bootstraps using the same package. Furthermore, Meirman’s *F’*_st_^67^ were calculated in the software GenAlEx 6.5^68^.

A Principal Component Analysis (PCA), using region as the grouping factor, was performed using the package Adegenet^69^. The principal components obtained from PCA were used as input synthetic variables to perform a Discriminant Analysis of Principal Components (DAPC) (for a full description of the method see Jombart, *et al.*^31^ and Horne, *et al.*^70^). The number of principal components to be retained in the discriminant analysis (N) was determined using alpha-score optimization, a method that finds a trade-off between discriminative power and model over-fitting.

To assess for the presence of spatial structure, we estimated genotypic spatial autocorrelation across three distance classes; 1) within single reefs 2) within the same geographic region 3) across the entire sampling area. We calculated individual based genetic distances in GenAlEx 6.5, using the codom-genotipic genetic distance option (for a detailed description of the method see Smouse and Peakall^71^). Autocorrelation coefficients (*r*)^71^,^72^ were calculated for each distance class in GenAlEx 6.5. We used 999 random permutations to generate 95% confidence intervals for the null model of no spatial autocorrelation, and 999 bootstraps to generate 95% confidence intervals of *r* for each distance class. The analysis was performed first for all sampled individuals, and then separately for males and females to detect possible sex-bias in spatial autocorrelation. The overall significance of each correlogram was tested using the non-parametric heterogeneity test outlined in Smouse, *et al.*^32^.

### Simulations

To determine whether the statistical analyses performed were capable of detecting genetic structuring at different levels of between-reef migration, we carried out a range of simulations using the software EASYPOP^73^. We created an 8 × 8 network of 64 reefs, with each reef encompassing an area of 20 km^2^. The census size of each reef was approximated based on the densities provided in the literature from pristine GBR reefs (2 sharks per hectare^24^). We assumed an effective population size (N_e_) to census size (N_c_) ratio of 1:10^74^, and created a dataset with the same number of loci and the locus diversity of the empirical dataset. Genetic drift was simulated for 100 generations with a 2-dimensional stepping stone model and levels of between reef migrations were set at 1%, 10% and 25%. Ten simulations were carried out for each migration level. We randomly sampled 10 individuals from three simulated reefs in each region of the simulated seascape, and carried out the same repertoire of analyses that were carried out for the empirical dataset. The value of each of the simulation parameters used was chosen to provide a conservative estimate of genetic drift: that is, the effect of genetic drift and the subsequent genetic structuring within the simulated dataset would be likely less than in our real data set (see Supplementary Material for details).

## Additional Information

**How to cite this article**: Momigliano, P. *et al.* Connectivity in grey reef sharks (*Carcharhinus amblyrhynchos*) determined using empirical and simulated genetic data. *Sci. Rep.* **5**, 13229; doi: 10.1038/srep13229 (2015).

## Supplementary Material

Supplementary Information

## Figures and Tables

**Table 1:** Sample sizes and genetic diversity indices.

**Region**	**N (mtDNA)**	**N_h_**	***h***	**N (msat)**	**A_r_**	**H_O_**	**H_E_**	**F_is_**
North GBR	32	9	0.8	32	6.93	0.8	0.78	−0.023
Coral Sea	8	3	0.61	8	5.6	0.79	0.75	−0.062
Central GBR	42	5	0.66	54	6.89	0.79	0.79	−0.002
South GBR	27	6	0.73	27	6.78	0.79	0.8	0.0124

N = sample size, N_h _= number of haplotypes, *h *= haplotype diversity, A_r _= allelic richness, H_O _= observed heterozygosity, H_E _= expected heterozygosity. F_is _= fixation index.
