# Supplementary material for: Connectivity in grey reef sharks (Carcharhinus amblyrhynchos) determined using empirical and simulated genetic data
Source: Sci Rep. 2015 Aug 28;5:13229. doi: 10.1038/srep13229 (PMC4551972; doi:10.1038/srep13229)
Supplement: Supplementary Information [file srep13229-s1.pdf]

Supplementary materials

Connectivity in grey reef sharks (*Carcharhinus amblyrhynchos*) determined using empirical and simulated genetic data.

.

Paolo Momigliano[1,2,]*, Robert Harcourt[1], William D. Robbins[3,4] and Adam Stow[1]

[1]Department of Biological Sciences, Macquarie University, Sydney, 2109 New South Wales, Australia

[2]Sydney Institute of Marine Science, 19 Chowder Bay Road, Mosman, 2088 New South Wales, Australia

[3]College of Marine and Environmental Science, James Cook University, Townsville, 4810 Queensland, Australia

[4]Wildlife Marine, Perth, 6020 Western Australia, Australia

* Corresponding author: paolo.momigliano@students.mq.edu.au

Simulation parameters:

To determine whether the statistical analyses performed would be able detect genetic structuring at different levels of between-reef migration, we carried out a range of simulations using the software EASYPOP[1]. EASYPOP is a software that allows simulation of genetic drift using a range of migration models, and is oriented towards the simulation of microsatellite loci. It allows the user to set the number of loci, locus diversity, migration models, as well as mutation rates to mirror empirical datasets.

We created a network of 64 reefs (8x8), each reef with an area of 20 km^2. The census size of each reef was determined based on the densities available from the literature from pristine reefs (2 sharks per hectare)[2]. We assumed an effective population size (N_e) to census size (N_c) ratio of 1:10 [3] , and created a dataset with the same number of loci and the locus diversity of the empirical dataset. Genetic drift was simulated for 100 generations with a 2-dimensional stepping stone model and levels of between reef migrations set at 1%, 10% and 25%. Ten simulations were carried out for each migration level. We randomly sampled from three simulated reefs in each region of the simulated seascape 10 individuals, and carried out the same repertoire of analyses that were carried out for the empirical dataset.

The values for each parameters were chosen so that the simulation would be overly conservative: that is, the effect of genetic drift within the simulated dataset would be likely much smaller than in a real scenario. For example, reef area was set to 20 km^2, while most reefs in the Great Barrier Reefs are much smaller[4], and the suitable habitat for grey reef shark is likely only a subset of the total area[5]. For example, the shark densities used to estimate the census size for each reef were based on abundances in the reef slope of pristine reefs while shark density in the back reefs are known to be lower [2,6]. This results in inflated Nc and Ne. As the effects of genetic drifts is inversely related to Ne, the rate of genetic drift (and therefore our ability to detect it) will be likely much smaller in the simulated dataset compared to the empirical data. The number of generations (100) assumes reef sharks only colonized the GBR about 1000 years ago. Again, this is a very conservative choice as it implies a very short time scale for genetic drift to act. Furthermore, we only created a network of 64 reefs, while the GBR contains > 3000 reefs, and we sampled only 10 individuals from 3 reefs in each region (a smaller sample size than our empirical dataset). Therefore, our power to detect genetic differentiation in the real dataset is likely to be much higher than in the simulated dataset.

Table S1 : Sampling locations and sample sizes (N). Note: as sex data were not available for all individuals, the sum of N males and N females is less than the total sample size N.

Location	Region	Latitude	Longitude	N	N males	N females
Day Reef	North GBR	-14,475	145.520	8	0	0
Lizard Island	North GBR	-14,677	145,444	24	12	12
Herald Cays	Coral Sea	-16,967	149,150	8	0	0
Barnet patches	Central GBR	-18,083	146,917	1	0	1
Pith Reef	Central GBR	-18,205	147,016	1	1	0
The Slashers	Central GBR	-18,539	147,121	1	1	0
Centipede Reef	Central GBR	-18,724	147,537	12	6	6
Lynch Reef	Central GBR	-18,777	147,710	14	2	5
Big Broadhurst Reef	Central GBR	-18,867	147,692	7	3	4
Judith Wright Reef	Central GBR	-18,893	147,950	6	3	3
Tobias Reef	Central GBR	-19,097	148,248	4	2	2
Darley Reef	Central GBR	-19,191	148,292	8	4	4
Heron Island reef	South GBR	-23,450	151,917	24	10	14
Wistari Reef	South GBR	-23.453	151.879	1	0	1
One Tree Island	South GBR	-23,489	152,065	2	0	2

Table S2: Microsatellite loci used in this study. N=sample size, N_A=number of alleles, H_0= observed heterozygosity, H_E= expected heterozygosity. The fluorescent dye used is indicated in superscripts: [1]6-FAM, [2] VIC®, [3] NED®, [4] PET®. Loci amplified using the M13 tail protocol[7] are indicated with *. All loci were isolated by Momigliano, et al. [8], with the exception of Cpl169 which was isolated by Portnoy, et al. [9]. PCR conditions were as described in Momigliano, et al. [8]

Locus	Primer Sequence (Forward and Reverse)	Motif	Size (bp)	N	N_A	H_O	H_E
C.amb2	F- TCCTACCTGACAAAGGAACTGC[4]<br>R- ATGAACAGAGACAAACAGACCGAC	$(TCTA)_{14}$	161-204	112	15	0.911	0.894
C.amb3	F- TGGAGTGCCAATTCTCTTGTCG[1]<br>R- ACTTGGGAGTCTGACTAATCTCC	$(TC)_{18}$	200-272	120	34	0.892	0.929
C.amb4	F- GTCGAATGCATTGAGTTTCAGG[2]<br>R- CCAATACAAGCAAAGGGACAAC	$(AC)_{14}$	344-380	118	16	0.771	0.814
C.amb5	F- CAGATATGCGGTGTCGTGGC[1]<br>R- TTCCGCGTCTTGTCTCTGC	$(TG)_{13}$	266-284	118	8	0.805	0.784
C.amb6*	F- TGTGGCTGGGATAAAATGCACG[3]<br>R- TGGCTTGTAAAATCCTGTTCTGCG	$(TGG)_{13}$	251-296	120	12	0.808	0.795
C.amb7	F- AGAATGCTGTCTCGTGATGC[3]<br>R- GTTGTCACTGTCGAGATAGAGC	$(AGAC)_{11}$	287-319	121	8	0.758	0.729
C.amb9	F- CCCAGGAGCCCTCTCTGTA[4]<br>R- GTCTCTTGCCACGCTCCTAC	$(TG)_{13}$	209-227	120	8	0.533	0.576
C.amb11	F- TGAACGCTTTACTGAACCTTGC[3]<br>R- GCAGCCTTTACTCCTCGTCA	$(CA)_{14}$	164-204	120	18	0.883	0.883
C.amb15	F- GTATGAGACGAGCATCGTGCC[2]<br>R- AATCGCAGCGTCTGCAATG	$(AC)_{13}$	192-230	119	14	0.882	0.828
C.amb18	F- TGCACACGCAGTGATGTTGG[2]<br>R- ATGCCGATTTCTCTGTTAATGAGC	$(AC)_{16}$	135-195	120	26	0.958	0.944
C.amb20	F- ATGTGGAGGAGTGATGTTAGCC[1]<br>R- TTAATGTCAGTGTTCACGCTGG	$(GT)_{12}$	306-354	119	17	0.891	0.906
C.amb22	F- ATGTCAGTTCTTTAGGAGTAGGG[1]<br>R- CCAATCTACACTTCACTCACTG	$(GA)_{11}$	346-356	118	3	0.356	0.308
C.amb25*	F- GACTCATCAGGATAGTCTGGATGCT[1]<br>R- GCTCAACTGTCAAAAGAGGAAGCC	$(AGGG)_8$	200-252	121	13	0.802	0.802
C.amb27*	F- AGTCAGTGTCACGATGG[4]<br>R- GCTTTCTATCATTAACATGAGATCC	$(TG)_{11}(AG)_{18}$	167-201	121	12	8.818	0.844
C.amb28*	F- CACATTGCTATGAGCCTGGAG[2]<br>R- CATCTCTTTCATCACTGCATGATTG	$(AC)_{13}$	286-348	118	15	0.746	0.799
Cpl169*	F- TGACACAACCATTTATTCCCACG<br>R- GGTTTCCTTGAGTGAAAGAGAGAGC	$(TG)_{42}$	118-198	118	33	0.890	0.944

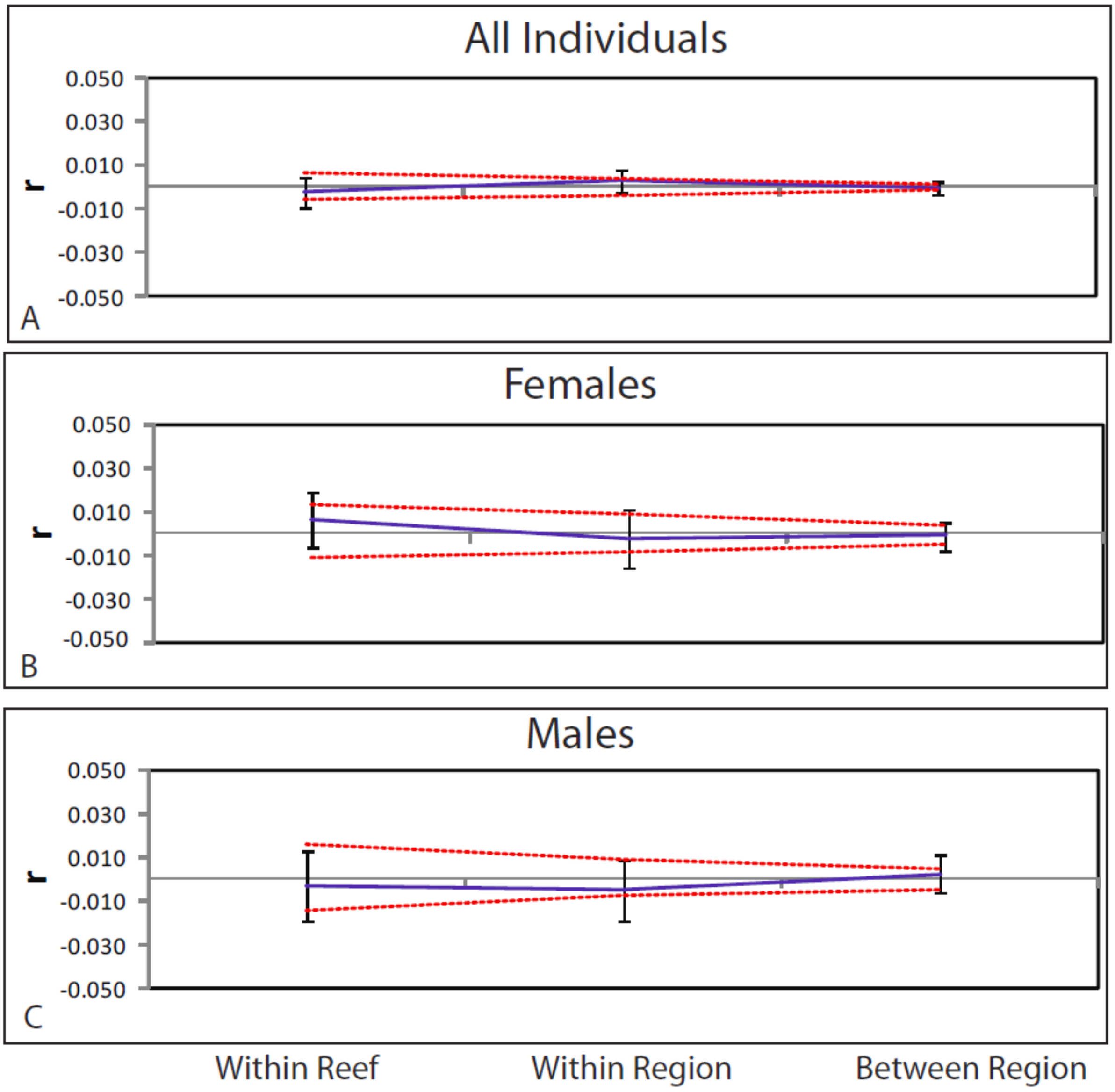

Figure S1: Genotypic spatial autocorrelation. Estimates of genotypic spatial autocorrelation (*r*) across different spatial scales. The x axis represent the different spatial scales (within a single reef, within a geographic region and across the entire sampling area), while the y axis represent the spatial autocorrelation coefficient (*r*). Analyses were carried out for all individuals (A) and then separately for females (B) and males (C). Error bars represent 95% confidence intervals estimated from 999 bootstraps. Red dotted lines represent the confident intervals of the null model (no spatial autocorrelation) estimated from 999 permutations.

References

1 Balloux, F. EASYPOP (version 1.7): a computer program for population genetics simulations. *J. Hered.* **92**, 301-302 (2001).

2 Robbins, W. D., Hisano, M., Connolly, S. R. & Choat, J. H. Ongoing collapse of coral-reef shark populations. *Curr. Biol.* **16**, 2314-2319 (2006).

3 Frankham, R. Effective population size/adult population size ratios in wildlife: a review. *Genet. Res.* **66**, 95-107 (1995).

4 Almany, G. *et al.* Connectivity, biodiversity conservation and the design of marine reserve networks for coral reefs. *Coral Reefs* **28**, 339-351 (2009).

5 Espinoza, M., Cappo, M., Heupel, M. R., Tobin, A. J. & Simpfendorfer, C. A. Quantifying shark distribution patterns and species-habitat associations: implications of Marine Park Zoning. *PloS one* **9**, e106885 (2014).

6 Rizzari, J. R., Frisch, A. J. & Connolly, S. R. How robust are estimates of coral reef shark depletion? *Biol. Conserv.* **176**, 39-47 (2014).

7 Schuelke, M. An economic method for the fluorescent labeling of PCR fragments. *Nature biotechnology* **18**, 233-234 (2000).

8 Momigliano, P., Robbins, W. D., Gardner, M. & Stow, A. Characterisation of 15 novel microsatellite loci for the grey reef shark (*Carcharhinus amblyrhynchos*). *Conservation Genetics Resources*, 1-3 (2014).

9 Portnoy, D., Mcdowell, J., Thompson, K., Musick, J. & Graves, J. Isolation and characterization of five dinucleotide microsatellite loci in the sandbar shark, Carcharhinus plumbeus. *Mol. Ecol. Notes* **6**, 431-433 (2006).
